# Merge in the Human Brain: A Sub-Region Based Functional Investigation in the Left Pars Opercularis

**DOI:** 10.3389/fpsyg.2015.01818

**Published:** 2015-11-27

**Authors:** Emiliano Zaccarella, Angela D. Friederici

**Affiliations:** ^1^Department of Neuropsychology, Max Planck Institute for Human Cognitive and Brain SciencesLeipzig, Germany; ^2^Berlin School of Mind and Brain, Humboldt-Universität zu BerlinBerlin, Germany

**Keywords:** pars opercularis, clusters, syntax, merge, fMRI

## Abstract

Language is thought to represent one of the most complex cognitive functions in humans. Here we break down complexity of language to its most basic syntactic computation which hierarchically binds single words together to form larger phrases and sentences. So far, the neural implementation of this basic operation has only been inferred indirectly from studies investigating more complex linguistic phenomena. In the present sub-region based functional magnetic resonance imaging (fMRI) study we directly assessed the neuroanatomical nature of this process. Our results showed that syntactic phrases—compared to word-list sequences—corresponded to increased neural activity in the ventral-anterior portion of the left pars opercularis [Brodmann Area (BA) 44], whereas the adjacently located deep frontal operculum/anterior insula (FOP/aINS), a phylogenetically older and less specialized region, was found to be equally active for both conditions. Crucially, the functional activity of syntactic binding was confined to one out of five clusters proposed by a recent fine-grained sub-anatomical parcellation for BA 44, with consistency across individuals. Neuroanatomically, the present results call for a redefinition of BA 44 as a region with internal functional specializations. Neurocomputationally, they support the idea of invariance within BA 44 in the location of activation across participants for basic syntactic building processing.

## Introduction

Traditionally language is thought of as one of the most complex cognitive functions. Recently, it has been claimed, however, that the human capacity to process complex syntactic structures is based on a very basic binary process which syntactically binds words together hierarchically to form larger structures. Because of the fundamental nature of this computation, called *merge* in theoretical linguistics (Chomsky, 1999; Adger, 2003), the determination of its neural implementation would constitute the neurobiological basis of a process which is at the root of any complex syntactic structure (Berwick et al., 2013). Up to now, the operation has almost never been directly studied in isolation, as syntax usually has been studied in more complex sentential contexts (Just et al., 1996; Stromswold et al., 1996; Moro et al., 2001; Cooke et al., 2002; Röder et al., 2002; Ben-Shachar et al., 2003, 2004; Constable et al., 2004; Bornkessel et al., 2005; Fiebach et al., 2005; Grewe et al., 2005; Friederici et al., 2006b; Santi and Grodzinsky, 2007, 2010; Caplan et al., 2008; Kinno et al., 2008; Newman et al., 2010). These and other studies across different languages indicate that the larger region in and around Broca's area in the inferior frontal cortex (IFG) supports syntactic processes (for reviews see Vigneau et al., 2006; Friederici, 2011).

A second region to be considered in the frontal cortex is the frontal operculum (FOP) which is a phylogenetically older than Brodmann Area (BA) 44 (Sanides, 1962; Friederici, 2006) and has been shown to be involved in syntactic classification as well as word based processing (Grasby et al., 1994; Stowe et al., 1998; Friederici et al., 2000b, 2006a). The adjacent anterior insula (aINS) is involved in the processing of short two-word sequences independent of whether they constitute a phrase or not (Zaccarella and Friederici, 2015). Other studies trying to localize syntactic processes reported the involvement of temporal regions, i.e., the left posterior superior and anterior temporal lobe (ATL) regions rather than inferior frontal regions (Bottini et al., 1994; Stowe et al., 1999; Vandenberghe et al., 2002; Humphries et al., 2005, 2006). These studies, however, used long sentences and compared these to word-lists often allowing minimal syntactic processes. The lack of any ATL activity in studies comparing sentences that only differed in syntactic complexity (Friederici et al., 2006b) speaks in favor of a compositional semantic role rather than a syntactic role of this area (Barsalou, 1982; Humphries et al., 2007). Indeed, a series of recent magnetoencephalography (MEG) experiments looking at conceptual compositionality effects at the phrasal level, found the ATL to be active during the construction of complex semantic representations, when color concepts had to be combined together with real objects, or when the same colors had to be combined with nouns (object labels) carrying semantic information (Bemis and Pylkkänen, 2011, 2013; Del Prato and Pylkkanen, 2014). Semantic sensitivity for the area is strongly confirmed by a recent fMRI study showing that activity change in the ATL varied as a function of the presence of lexico-semantic information, but not of syntactic variables, during the construction of progressively increasing linguistic structures (Pallier et al., 2011).

The goal of the present study was to identify the neural basis of the most basic syntactic computation, upon which any more complex hierarchical structure can be derived. By this computation two words, i.e., *this* and *ship*, are bound together to a hierarchical phrase containing both words—i.e., *this ship* with *this* dominating *ship*. By applying the same mechanism again, we then recombine this phrase with the closest element occurring in the sentence, to form increasing syntactic hierarchies—i.e., *this ship sinks*. Phrases of two-word length—like *this ship*—are the ideal level to investigate this most basic process of syntactic binding, as the amount of cognitive load required to process such small constructions is very limited. This means that—after classifying *this* as a determiner and *ship* as a noun—only the operation of merge is necessary to make it a phrase. Crucially, at the very same two-word level, it is also possible to create contexts consisting of simple lists of words—like *stone, ship*—in which no phrase can be created, as no syntactic relationship holds. Because of this minimal opposition, two-word manipulations are an ideal level to identify the most basic and essential syntactic computation of *merge*. We hypothesize that this computation should be located in Broca's area, as this region has been found to support syntactic processes (Vigneau et al., 2006; Friederici, 2011; Hagoort and Indefrey, 2014) and further that if its assumed fundamental nature holds, it should be localizable (a) in a very confined subregion within this area, and (b) with little variance across individuals.

Neuroanatomically, Broca's area can be subdivided into BA 45 and BA 44, whose borders were first mapped based on the cellular organization of their regional tissues (Brodmann, 1909), and then redefined using observer-independent cell density profiles over histological slices of postmortem brains (Amunts et al., 1999). More recently, a multireceptor-based analysis separated BA 44 into an anterior dorsal part, and a posterior ventral part (Zilles and Amunts, 2009; Amunts et al., 2010). A very recent meta-analytic functional connectivity-based parcellation (CBP) approach even proposed a decomposition of BA 44 into five separate subregions, called clusters (Cs) two of which located in its more posterior part (C1 and C4), another two in the more anterior part (C2 and C3), and a third one in the inferior frontal junction (C5; Clos et al., 2013). The CBP approach first identifies the whole-brain co-activation pattern for each voxel contained in BA 44 across several thousands fMRI studies, and then groups together those voxels into distinct clusters, according to the similarity of their co-activation patterns across the brain. While the functional specificity of these five clusters as indicated by the metaanalysis is low, with each of the clusters' functional domain ranging from action, working memory, switching to other cognitive tasks, the fine-grained subdivision of BA 44 may allow a precise localization of the most fundamental syntactic process assumed for any natural language. Here we hypothesize, that if the high functional specificity of *merge* as a fundamental syntactic computation holds, this mechanism should to be localizable in one of the sub-clusters within BA 44, with little inter-individual variance.

This hypothesis was investigated in an fMRI study using two-word sequences either allowing hierarchical syntactic binding to apply (phrase trials) or not (list trials), which were presented visually. Phrase trials and list trials were constructed as parallel as possible, only varying in the possible application of syntactic binding. They differed in their first element, which was either a determiner (e.g., *this*) or a noun (*apple*), while the second element was always a phonotactically legal pseudoword (*flirk*) to drastically reduce conceptual-semantic processing in both conditions (Bemis and Pylkkänen, 2011). Two corresponding one-word conditions were also included, in which the pseudowords were substituted with a series of X's (e.g., *XXXXX*) to explore the effect of number of words. Thus, the experiment included two factors: type of STRUCTURE [“phrase” (PH), allowing syntactic binding, vs. “list” (LS) not allowing syntactic binding] and number of WORDS (“2-words” vs. “1 word plus Xs”). We employed three progressively region-stringent levels of data analysis to localize syntactic binding: (i) a whole-brain analysis to know whether BA 44 and FOP and/or the aINS show activity during two-word processing; (ii) a more restricted volume-of-interest analysis for BA 44 to directly contrast phrase processing vs. word-list processing; and (iii) a cluster-of-interest analysis within BA 44 to localize syntactic binding at the individual subject level. These three analyses allow us to test whether: (i) BA 44 is highly sensitive to structure formation even at the lowest level of phrase structure building; (ii) the fundamental nature of syntactic binding is expressed by a stringent localization in a subregion within BA 44, using a cluster-based approach in which the five clusters by Clos et al. (2013) are used to test sub-regional sensitivity for merge within BA 44; (iii) the invariant character of syntactic binding assuming little variance of the localization within this subcluster of BA 44 across individuals.

## Materials and methods

### Participants

We tested 27 right-handed subjects (Oldfield, 1971), but only 22 subjects (11 female; mean age 28.5 years, standard deviation (SD) 3.62 years; all native German speakers) were included in the analysis. Four subjects were excluded because of poor behavioral performance. One additional subject was excluded because the trial list file was corrupted. The local ethics committee of the University of Leipzig approved all procedures used during the experiment. Written informed consent was obtained from all subjects.

### Stimuli construction

At the 2-words level, phrasal syntactic contexts (2-PH) comprised eight adjectival determiners of two-syllables in length to appear as first word—*jede/jedes* (each)*, eure/euer* (your)*, jene/jenes* (that)*, diese/dieses* (this) followed by 48 different pseudowords (e.g., DIESE FLIRK). List contexts (2-LS) comprised eight nouns selected from the CELEX corpus for German (Baayen et al., 1995) which were matched to the determiners for syllabic length, letter length, and syllabic stress—*Apfel* (apple), *Käse* (cheese), *Ofen* (oven), *Efeu* (ivy), *Motor* (motor), *Kiwi* (kiwi), *Haken* (hook), *Koffer* (suitcase). A corresponding example of list context was APFEL, FLIRK. These words and the pseudowords were constructed and controlled according to all relevant psycholinguistic parameters, and for the pseudowords we strongly avoided associative effects with real words by using an automatized screening procedure based on the same CELEX corpus, followed by a final filtering selection done by three mother-tongue German speakers (see Appendix A–Stimuli construction for detailed information). Importantly, the use of pseudowords in either phrasal or list contexts at two-word level, was directly intended to reduce potential interactions due to semantic activity, given indeed that we were explicitly interested in finding neural correlates of syntactic processing in the brain. In doing so, we took advantage of the intrinsic linguistic distinction between the syntactically prominent functional lexicon (e.g., determiners, prepositions, conjunctions) and the semantically prominent contentive lexicon (e.g., nouns, verbs, adjectives), and coupled it together with the semantic-free nature of the pseudowords themselves. In this respect, since determiners have less semantic content that do nouns, the choice to use pseudowords instead of real words helped us to: (1) keep syntactic activity at work in determiner-pseudoword contexts, while removing semantic information; (2) remove syntactic and semantic information in the noun-pseudoword contexts, by reducing compounding effects via head de-lexicalization (see Supplementary Material); (3) shield further light on the role of BA 44 as syntactic-sensitive area. Finally, at the 1-word level, the pseudowords were substituted with a series of X's (e.g., XXXXX) to obtain 1-word phrasal contexts (1-PH: DIESE XXXXX) and 1-word list contexts (APFEL XXXXX).

### Procedure

Before entering the scanning room, participants performed a short practice session of the actual experiment on a desktop computer located just outside the MR unit area. None of the stimuli used in the instruction session were used during the experimental session. Once in the scanner, stimuli were presented visually using the software package Presentation® (Neurobehavioral Systems, Inc., Albany, CA, USA) with a Sanyo PLC-XP50L LCD XGA (Sanyo Electric Co., Ltd., Moriguchi, Japan; pixels = 1024 × 768; refresh rate = 100 Hz) back-mirror system mounted on the head-coil. Although the projector was already adjusted for minimal luminance, a white font/gray background was used, and preferred by all subjects. A mono-spaced font (Courier) was used (capitalized letters; 45 pt.). A single trial consisted of a white fixation cross which remained at the center of the screen until a random jitter of either 0 or 1000 ms after volume acquisition started the visual stimulation. Stimulus-onset-asynchrony was 8.6 s on average. All trials had a total duration of 900 ms and the items were presented sequentially on the screen one after the other (Supplementary Figure 1). Given that our stimulus construction was syllable-constrained, the first bi-syllabic word remained on the screen for 600 ms, while the second monosyllabic word/X string lasted 300 ms. As soon as the fixation cross reappeared, immediately after the second item within the trial had been shown, subjects were requested to perform a simple sequence judgment task similar to the one used in Friederici et al. (2000a), as quickly as possible, by indicating via triple-choice button-pressing whether the two words together formed a phrase (e.g., DIESE FLIRK = yes), whether they did not form a phrase, but just a list of two nouns (APFEL FLIRK = no), or whether it was a trash trial with X strings (DIESE XXXXX/APFEL XXXXX = trash). We used a fully counter-balanced stimulus exposure across conditions, such that in half of the cases the determiner (or the noun) was followed by a pseudoword, and in the other half of the cases by a sequence of Xs. Therefore, subjects could not discriminate between conditions on occurrence of the first word of the trial, rather they were forced to pay attention to the second word to solve the task. Subjects were requested to use the right index finger, the right middle finger, and the right ring finger to accomplish the task. The order of both buttons and trials were fully randomized across subjects. Each experimental dataset collection lasted ~42 min.

### Behavioral data analysis

Mean reaction times for correct responses (RTs) and accuracy rates were calculated for each condition of each participant and were analyzed using a Two-way within-subject analysis of variance (ANOVA), with factors STRUCTURE (phrase: PH vs. list: LS) and number of WORDS (2- vs. 1-word). Missing responses were counted as non-correct responses.

### fMRI data acquisition

Functional images were acquired with a 3T whole-body Bruker Medspec 3000 Scanner. The functional data were acquired using a T2^*^-weighted gradient-echo echo-planar-imaging (EPI) sequence, with the following parameters: TR = 2.0 s, TE = 30 ms, flip angle = 90°, FOV = 19.2 × 19.2 cm^2^, in-plane resolution = 3 × 3 mm^2^; data matrix = 64 × 64; slice thickness = 3 mm; interslice gap = 1 mm; number of slices = 30 (axial slices, parallel to AC-PC line/whole-brain coverage, ascending direction), number of volumes = 1270 volumes. T1-weighted 3D MP-RAGE (magnetization-prepared rapid gradient echo) images (Mugler and Brookeman, 1990)—TI = 650 ms; TR = 1300 ms; alpha = 10°; FOV = 256 × 240 mm—were previously acquired with a non-selective inversion pulse to be used for preprocessing of the functional data.

### Functional imaging data analysis

Functional data were analyzed using the SPM8 software package (http://www.fil.ion.ucl.ac.uk/spm/). In the pre-processing session, subject-specific functional volumes were co-registered with corresponding structural T1-weighted images. Functional time series were further realigned to the first image to correct for motion artifacts, and resliced for timing correction. A gray-matter segmentation-based procedure was used for normalization to the standard MR template included in the SPM software package. A Gaussian filter of 8 mm^3^ FWHM was used to smooth the data. A high pass filter of 128 s was used to attenuate slow global signal changes. These data entered in a number of analyses described below, they were also used for an additional analysis focusing on the insula and its subregions presented in a separate article (Zaccarella and Friederici, 2015).

#### fMRI whole-brain data analysis

The SPM8 software package was then used to perform a two-stage random-effects analysis to ensure result generalizability over the population level (Penny and Holmes, 2004). The first five volumes from each dataset were excluded to allow for magnetic saturation effect. Subject-specific general linear models were assessed using the hemodynamic response function from the SPM software (Friston et al., 1995). Single stimulus functions were modeled according to their timing onsets. Error trials and fillers trials were modeled as distinct conditions, and movement parameters were treated as regressors of no interest. Contrast estimates for the four experimental conditions (compared against the global mean) were obtained using first-level statistics. The contrast estimates were then used in a second-level within-subjects ANOVA to assess group contrasts. Statistical inferences were drawn at *P* < 0.05, with a Family-Wise Error (FWE) correction.

#### Volume-of-interest analysis

Following our initial hypothesis that BA 44 is responsible for merge processing, we focused on the left infero-frontal region alone, by performing a finer-grained analysis in BA 44 to assess the specific effect of phrases compared to lists, directly (2-PH > 2-LS). The cytoarchitectonically-defined BA 44 from the maximum probability map (MPM; Supplementary Figure 2) of the SPM Anatomy Toolbox (Eickhoff et al., 2005) served as an independent search space to avoid selection bias (Kriegeskorte et al., 2009; Vul and Kanwisher, 2010). A small-volume correction (SVC) was used to threshold the results, at *P* < 0.05, FWE-corrected.

#### Cluster-of-interest analysis

The multi-modal CBP map for BA 44 proposed in Clos et al. (2013) served as mask for the Cluster-of-Interest (COI) analysis. Here we first wanted to simply localize the cortical distribution of the active voxels to the contrast 2-PH > 2-LS, which we found at *P* < 0.05, FWE-corrected in the SVC analysis discussed above. This map, which is bounded by the same cytoarchitectonic region (MPM) that was used in the above SVC analysis, consists of five sub-regional BA 44 clusters comprising a posterior-dorsal cluster (C1), an anterior-dorsal cluster (C2), an anterior-ventral cluster (C3), a posterior-ventral cluster (C4), and an inferior frontal junction cluster (C5; see also Supplementary Figure 3). For our purposes, the activation mass obtained from the SVC analysis was first transformed into a binary image of zeroes (not-active voxels) and ones (active voxels), and then dot-multiplied with each cluster volume from the BA 44 parcellation map described above. Following this procedure we then counted the total number of ones (active-voxels) falling within each cluster to determine the overlapping region. Additionally, the five CBP clusters were further used as seed sub-regions to extract signal intensity, to evaluate the mean activity distribution of the syntactic binding effect across the different clusters. Mean signal extraction from the five clusters was done using Marsbar 0.41 for SPM (available at http://marsbar.sourceforge.net).

#### Individual peak activity distribution analysis

Finally, we were interested in assessing whether at the individual level, the peak distribution of neural activity across individual subjects was homogeneously spread within BA 44, or rather gathered around one of the CBP clusters described above, therefore showing little variance over space. To evaluate cluster sensitivity at the subject level, we again used the map for BA 44 as a binary searchable space, and dot-multiplied it with the subject-specific contrast we obtained as {T} maps from first-level statistics, for the contrast (2-PH > 2-LS). For each subject, we then extracted a unique 3D coordinate maximum corresponding to one voxel. Each 3D coordinate was in turn localized as belonging to a particular cluster, using each BA 44 sub-regional cluster as independent mask, following an analogous counting procedure of the one described in the COI analysis above. From the resulting distribution, we first performed a standard chi-square distribution test. We then employed a randomization test of goodness-of-fit to strengthen, or possibly weaken, the significance of our cluster-sensitivity. We therefore drew 10,000 random samples from a population with our known proportions we obtained from the data, re-calculated the chi-square for each replicate sample, and then counted how many times a larger chi-square value was obtained during randomization (McDonald, 2009). The proportion of replicates with chi-square values equal to or greater than the first observed value was then taken as final *p*-value. A threshold of *p* = 0.05 (5% of times) was chosen.

## Results

### Behavioral results

No significant effect for accuracy was found. A significant effect for STRUCTURE [*F*_(1, 21)_ = 25.003; *p* < 0.0001] and an interaction between WORDS and STRUCTURE [*F*_(1, 21)_ = 5.896; *p* < 0.05] were found for the reaction time data. A series of paired *t*-tests revealed that subjects were slower for 2-LS compared to 2-PH (*t* = 3.93; *p* < 0.001). An almost significant difference for LS: 2 > 1 was found (*p* = 0.059), while there was no significant difference for the contrast PH: 2 > 1 (*p*>0.1; Supplementary Figure 4).

#### Whole-brain analysis

We found a main effect of WORDS in the left and right FOP/adINS at *x* = −33; *y* = 23; *z* = −2 and *x* = 36; *y* = 23; *z* = −2, respectively, and in the left BA 44 at *x* = −48; *y* = 11; *z* = 7. The main effect of STRUCTURE, as well the WORDS × STRUCTURE interaction did not yield any significant clusters that survived standard statistical thresholds (Supplementary Table 1).

#### Volume-of-interest analysis

**Figure 1:**
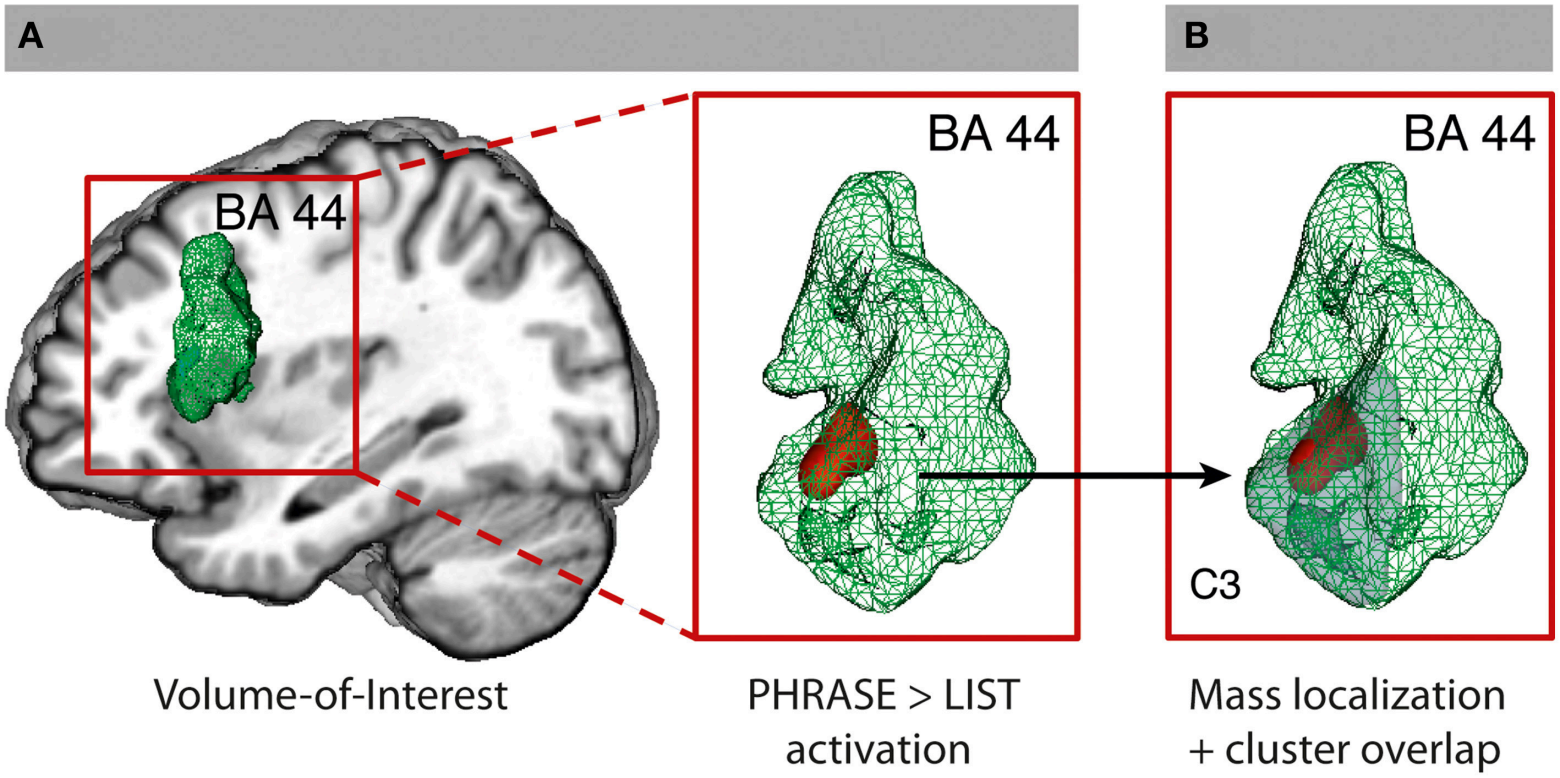
**Volume-of-interest analysis for BA 44**. **(A)** BA 44 VOI from the SPM Anatomy Toolbox and SVC (FWE-corrected *P* < 0.05) for the contrast PHRASE>LIST at two-words level (2PH > 2-LS). **(B)** C3-cluster (Clos et al., 2013; light gray) with overlapping SVC activation. See also Supplementary Figures 2, 3.

The hypothesis-driven analysis in BA 44 revealed a significant cluster for phrase compared to list at two-words level (2-PH > 2-LS) in the ventral anterior part of BA 44 at *x* = −48; *y* = 17; *z* = 16, using a small volume correction analysis (SVC) in the area. No significant voxel was found active for the opposite contrast (2-PH < 2-LS), even at more liberal thresholds. To note, prior direct comparison between phrase and list at two-words level, we run an ANOVA with factors WORDS and STRUCTURE within BA 44 to gain information about a possible interaction within the region. Interestingly, we detected within the region the 3D voxel showing the highest peak by downloading the unthresholded WORDS × STRUCTURE activation map we obtained from the SPM group-averaged output (*x* = −51; *y* = 20; *z* = 13). From this 3D coordinates we then extracted signal intensity for all four conditions to verify whether an interaction between WORDS and STRUCTURE would have survived statistical control. To note, we found a significant interaction between the two factors at *p* = 0.039 level [*F*_(1, 21)_ = 4.83]. Direct comparison between 2-PH and 1-PH was significant at *p* < 0.001 level (*t* = 3.87), as it was between 2-PH and 2-LS at *p* = 0.007 level (*t* = 2.94). Direct comparison between 2-LS and 1-LS was not significant (*t* = 1.67; *p* = 0.11; see Figure 1A. See also Supplementary Table 1 and Supplementary Figure 5 for the interaction effect). We further performed additional independent analyses in the other regions that were found to be active for the main effect of WORDS, to evaluate whether syntactic binding was specifically performed in BA 44 alone, or whether additional portions of the cortex were also involved. There was no difference between phrases and lists in the other regions under analysis (see also Appendix B–Volume-of-interest Analysis for more information). To gain further exploratory indication on the relative functional contribution of phrases and lists to the main effect of WORDS in the inferior frontal regions, and to verify the results we obtained from the SVC analysis, we went then back to our full brain datasets and performed two distinct planned contrasts at the two levels of the STRUCTURE factor. For the contrast lists vs. one-word list condition (LS: 2 > 1) we found activity in the opercula only at *x* = −33; *y* = 23; *z* = −2 and *x* = 36; *y* = 23; *z* = −2. For the contrast phrases vs. one-word phrase condition (PH: 2 > 1) we found additional recruitment of the ventral-anterior portion of left BA 44 at *x* = −51; *y* = 11; *z* = 7, which, together with the LS: 2 > 1 results, suggests a stronger involvement of BA 44 for phrases than for lists. At the whole-brain level, however there was no significant difference in activity for the contrast phrases and lists (2-PH > 2-LS) using standard threshold methods.

#### Cluster-of-interest analysis within BA 44

**Figure 2:**
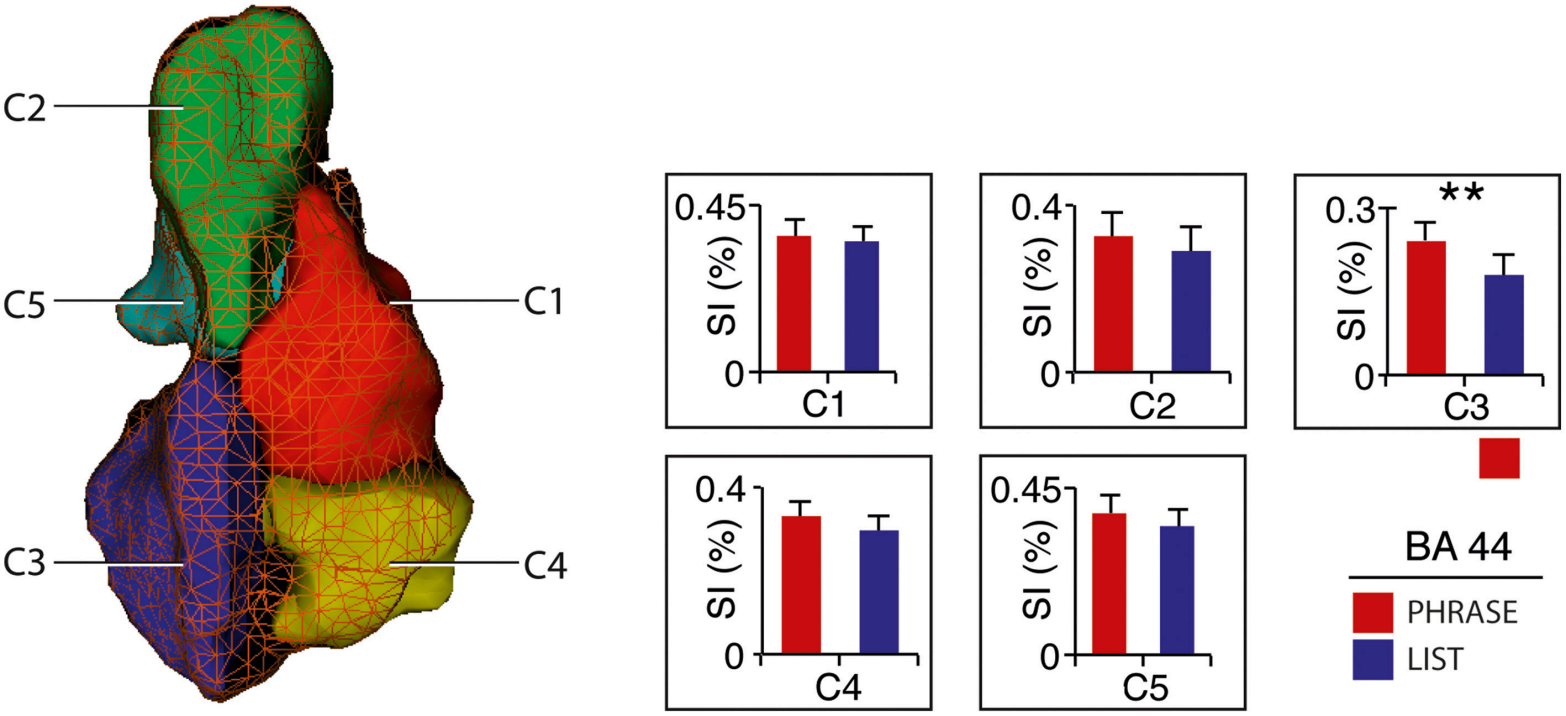
**Activity distribution within BA 44 – Cluster-of-interest analysis**. Signal intensity for 2-word-phrases and 2-word-lists with clusters (Clos et al., 2013). Error bars denote SEM. ^**^p < 0.01. See also Supplementary Figure 6.

All voxels active for the contrast phrase vs. list at two-words level (2-PH > 2-LS) in the SVC in BA 44 fell within the anterior–ventral cluster C3 (100% overlap; 12/12 voxels; see Figure 1B). Remarkably, paired *t*-tests for signal intensity revealed a significant difference in activity between phrases and lists (2-PH > 2-LS) in the C3 cluster only [*t*_(21)_ = 2.97, *p* = 0.007, surviving Bonferroni-correction for the number of tests [*p* = (0.05/5 tests) = 0.01], and in no other sub-region (see Figure 2 and Supplementary Figure 6).

#### Individual peak activity distribution analysis

**Figure 3:**
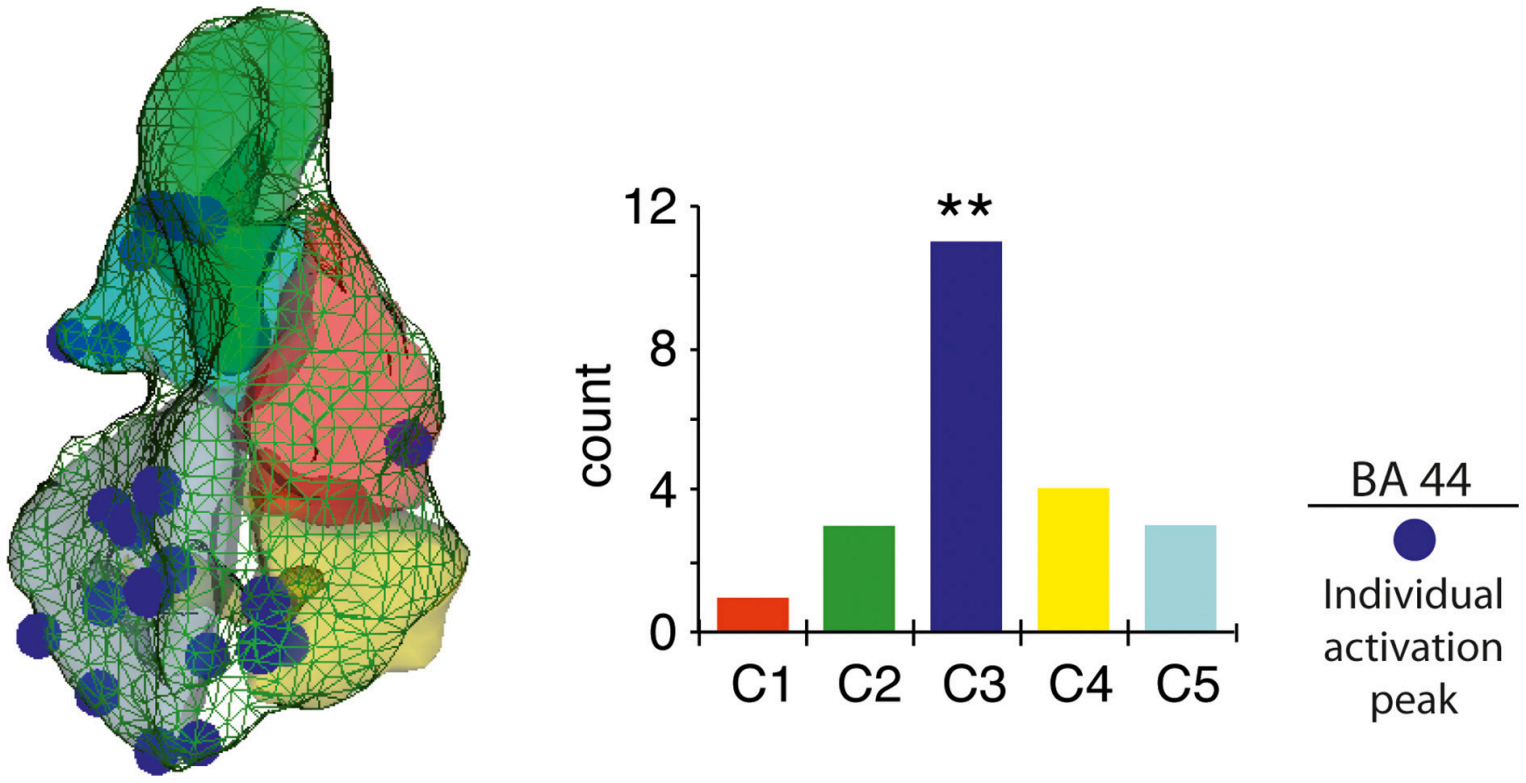
**Individual peak activity distribution within BA 44**. Unique 3D coordinates maximum peak localization for 2-word-phrases versus 2-word-lists (left), with vertical histogram reporting frequency distribution per cluster (right). ^**^p < 0.01.

A strong cluster-sensitive distribution of the individual peak activity for the contrast (2-PH > 2-LS) was found in C3, as compared to the other BA 44 sub-regions [$\chi_{\left(4\right)}^{2}=13.45$, *p* = 0.009, confirmed after 10,000 randomization tests for goodness-of-fit; see Figure 3].

## Discussion

The goal of the present fMRI study was to identify the neuroanatomical basis of the most fundamental syntactic computation, which is at the root of all natural languages (Chomsky, 1995; Berwick et al., 2013). This basic computation, called *merge*, which binds two words together syntactically, allows to build up syntactic structures with increasing hierarchy. Here we found that this most basic process of syntactic binding corresponded to increased activity in a most confined brain region, i.e., the anterior section of the ventral left pars opercularis, BA 44 at the posterior part of Broca's area. Conversely, a phylogenetically older area, the FOP/adINS, was found to be equally active for both phrasal structures and unstructured word-lists, not discriminating between these.

With respect to the FOP/aINS, the present analysis indicates that this area's function—previously identified by a region-of-interest analysis of the insula—is also identifiable at the whole brain analysis, thereby extending results from the same study (Zaccarella and Friederici, 2015). Its involvement revealed from the contrast between two- and one-word stimuli may reflect word-accumulation processes during which the categorical information and the grammatical status of the word is first accessed (Friederici et al., 2000b) and then shortly maintained on hold (Grasby et al., 1994), before further processing takes place. A similar activity pattern found for both phrases and lists is not surprising, given the low degree of functional specialization that the FOP/adINS has inside the language processing system (Saygin et al., 2004; Mutschler et al., 2009). This lower specialization of the left FOP/adINS compared to BA 44 also finds support in another fMRI study which showed that this area, in contrast to BA 44, was not able to distinguish between grammar types, but only able to detect an error in the order of syllables in sequences (Friederici et al., 2006a).

In BA44 we found that the basic operation of syntactic merge was sensitive to a specific cluster within the region, such that only the anterior-ventral cluster as one among five sub-regions discriminated between phrases and lists. Crucially, we discovered that the localization of this activity within the same anterior-ventral cluster was highly consistent across participants. While a previous functional connectivity-based meta-analysis described this subregion as being associated with all kinds of language processes (Clos et al., 2013), the present study is the first to delineate this subregion from other regions within BA 44 in its function in language. This particular subregion appears to be responsible for the most basic syntactic computation at the root of all syntactic hierarchies. Remarkably, within neurolinguistic literature, the contribution of parts of Broca's area, in particular BA 44, to syntactic processing has mostly been discussed in terms of syntactic complexity at the sentential level, since the area was found to be crucial for the processing of syntactically more complex sentential hierarchies, compared to simpler ones (Röder et al., 2002; Friederici et al., 2006a,b; Bahlmann et al., 2008). The present data are in line with the view of BA 44 being activated as a function of structural hierarchy, *but* they clearly go beyond this view by demonstrating that the most basic syntactic computation upon which more complex hierarchies are built, can be neuroanatomically located in a sub-region of BA 44. This means that because both complex and simpler linguistic hierarchies necessarily share the same computational merging algorithm, BA 44 activates as a function of structural hierarchy regardless of the linguistic complexity itself.

The sustained cluster-sensitivity of the merge computation in the anterior-ventral cluster of BA 44 both at the group and the individual level points toward a fundamental and constrained nature of *merge* at the neural level. While we acknowledge that the present work only tested for one single language, the low interindividual spatial variability we found across our representative set of subjects, might be taken as a first approximate indication in favor of the fundamental character of the computation itself. The present finding of a sub-regional specificity and invariability of the most basic process closely resembles the neural organization of other basic sensory processes, as for example in the visual system (Downing et al., 2006), while the low inter-individual variability of the basic processes gives rise to the assumption that their function to structure relation is predetermined. In this respect, because of the essential nature of merge as being the shared computation across all human languages, future studies should systematically focus their attention on the neural implementation of very basic linguistic processes in multiple languages. This would ultimately prove whether the universality of merge at theoretical level (Chomsky, 1995) adequately corresponds to some neuroanatomical generalizability at the neural level suggested here.

From an evolutionary perspective, no clear evidence for a human-like language syntax in nonhuman species has been presented so far (Fitch and Hauser, 2004; Bolhuis et al., 2014). Structural studies have shown that the FOP is a phylogenetically older cortex, which is fully represented in monkeys (Sanides, 1962), while BA 44 seems to be more expressed in humans than in monkeys, in which it plays a role in orofacial somatomotor processes (Petrides et al., 2005). The activation pattern we report in our study closely resembles the one proposed for human and non-human artificial grammar processing in the adult brain, in which violations to transition probabilities are found to activate FOP, while violations to more rules activate BA 44 (Friederici et al., 2006a). This functional split between the labor of FOP/aINS and that of BA 44 is particularly intriguingly if put into relation with recent theoretical linguistic models, which propose that the specificity of merge in language should reside in the property that words have to create constituents where the lexical label of the single dominant word (e.g., determiner) is reflected as a hierarchical influence onto the newly created syntactic constituent (e.g., determiner phrase; Boeckx, 2010; Chomsky, 2013; Murphy, 2015). At the interface between linguistic theory and neurolinguistics, the merge mechanism would then consist of two phases: one in which linguistic elements are strung together without any hierarchical dimension in the FOP/aINS, and a labeling phase in which the dominant lexical element transforms the string into a hierarchically labeled syntactic structure in the anterior-dorsal BA 44. We believe that this speculation can constitute a testable model for the evolution of the language faculty, in which behavioral, functional and anatomical data can be put together in a comparative perspective within across human and animal species (Murphy, 2015).

## Conclusion

The sub-anatomical specificity for the process of syntactic binding, called *merge*, has a strict neural basis in the anterior-ventral cluster of BA 44. The profoundly constrained regional localization of this syntactic operation converges on the conclusion that the computation at the root of our syntactic knowledge has strict neural basis. The constraint localization of the activity and its consistency across the participants point toward the fundamental neurobiological nature of the operation of *merge* itself, thereby providing a novel view on the relation between linguistic theory and neurobiology.

### Conflict of interest statement

The authors declare that the research was conducted in the absence of any commercial or financial relationships that could be construed as a potential conflict of interest.
