# Supplementary material for: Merge in the Human Brain: A Sub-Region Based Functional Investigation in the Left Pars Opercularis
Source: Front Psychol. 2015 Nov 27;6:1818. doi: 10.3389/fpsyg.2015.01818 (PMC4661288; doi:10.3389/fpsyg.2015.01818)
Supplement: Supplementary file 1 [file DataSheet1.PDF]

Appendix A

Stimuli construction

Forty-eight monosyllabic pseudo-words were created to appear as the second word in both 2-word level conditions. The choice to use pseudo-words instead of real words was motivated by the fact that German language has highly productive compounding mechanisms, such that almost any N-N pair would have possibly produced conflicting compositional effects with the opposing phrasal syntactic condition. Given that German is a head-final language with left-branching compounding procedures, the last word in noun-noun pairs constitutes the head which semantically defines the compound and which carries relevant lexical traits (i.e., grammatical gender and number) to be expanded to all its preceding modifiers (i.e., the preceding nouns). We therefore reasoned that head de-lexicalization could have been a profitable way to inhibit lexical compounding in the list condition and concurrently avoid conceptual integration activity in the brain. The WordGen (Duyck et al. 2004) program (http://expsy.ugent.be/wordgen.htm) was used to generate the pseudo-words. We decided to use the WordGen program because it assembles strings of randomly selected letters in an automatized fashion, and interactively verifies their existence as real words in the lexical database of a specific language. Additionally, we could add further efficiency by using some important constraints to control for string generation, such as letter length, neighborhood size and forbidden letter lists, together with a bigram occurrence analysis we performed prior to final selection. For our purposes, the CELEX corpus for German was automatically set as default lexical database. We first selected pseudo-words of only four and five letters because this is most consistent with the relative letter length distribution that monosyllabic words show in German language. Words of four or five letters indeed represent approximately 70% of all monosyllabic words listed within the CELEX corpus, with four-letter words being about two-thirds of the relative total (total number of words found = 1306 | four-letter words = 552 (42.26%) | five-letter words = 344 (26.33%) | relative total = 896 (68.59%) with relative four-letter words = 61.60% and relative five-letter words = 38.39%). With respect to the lexical neighborhood size, this constraint expresses the amount of real orthographic neighbors that a pseudo-word can have by changing one single letter within the word. This value is also known as the *N*-metric, or Coltheart's *N* (Coltheart et al. 1977). There exists consistent evidence in the behavioral literature showing that pseudo-words are categorized quicker if they resemble a relatively low number of real words, than if they resemble a relatively large sample, as for example shown in lexical decision tasks (Andrews 1997). In parallel, recent neurophysiological evidence has proven that the processing of pseudo-words with relative

low N-metric generates smaller N400 effects in ERP recordings, compared to the processing of pseudo-words with many real neighbors (Holcomb et al. 2002). Since we wanted to avoid uncontrolled lexical access/associative processing during scanning acquisition as much as possible, and therefore reduce functional unexpected variance in the brain, we asked the program to only select pseudo-words with a very low N-metric value of N = 2. Finally, we also avoided forbidden letter lists by employing a two-step screening-exclusion procedure, the former internal to WordGen, and the latter implemented after the final WordGen list was obtained. We first asked the program to exclude all possible endings carrying morphological information—such as verbal and lexical inflections—that could have misled categorical NP interpretation. We then wanted to be sure that all two-letter bigrams presented in our pseudo-word set—irrespective of position—were possible bigrams that could potentially be found in the set of monosyllabic real words listed in the CELEX corpus. Therefore once the final pseudo-word set from WordGen was obtained, we filtered the list by excluding all those pseudo-words showing not-attested bigrams from the CELEX set of monosyllabic words. This procedure also helped to exclude impossible German bigram occurrences. Finally, three mother-tongue speakers read the list and excluded pseudo-words with remaining moderate associative effects. We ended with thirty-four four-letter long pseudo-words (70.83%), and fourteen five-letter long pseudo-words (29.16%). The stimulus pool consisted of 192 trials, with 48 trials each for each of the four conditions: 2-PH (DIESE FLIRK); 2-LS (APFEL FLIRK); 1-PH (DIESE XXXXX); 2-LS (APFEL XXXXX). To perfectly equalize the total number of visual occurrences between bi-syllabic words (192 times across the four conditions) and monosyllabic pseudo-words (96 times across the 2-word conditions), we also included 96 trash filler items in which we reversed 1-word conditions stimuli by replacing each bi-syllabic word with X strings of equal length, and by keeping the pseudo-words visible (i.e., XXXXX FLIRK). These fillers were matched with 48 full X string trials in which both the first and the second word were substituted with X strings to limit subjects' second word expectancy on the filler items. Half of the pseudo-words used in the filler items were taken from the experimental 2-word conditions, half of them were created ex novo using the same procedure described above for the pseudo-words creation.

Appendix B

Volume-of-Interest Analysis

The masks for the SVC analyses were defined on the basis of the Harvard-Oxford Cortical (HOC) atlas (Desikan et al. 2006) that accompanies FSL (25% probability; MNI152 space).

Besides the fact that—compared to the Anatomy Toolbox—the atlas covers the entire cerebral cortex, this choice was based on the fact that activity in the insular complex was found in its anterior portion only, and recent cross-modal parcellation maps based on the HOC atlas have indeed found that the region can be split into an anterior-dorsal cluster, a more middle cluster, and a posterior cluster, based on task-evoked co-activation, intrinsic (i.e., task-independent) functional connectivity, and gray matter structural covariance (Kelly et al. 2012). Since these clusters are freely available on the internet (http://fcon_ 1000.projects.nitrc.org), we were able to constrain the analysis to the anterior insular cortex alone, also based on previous literature stating that within the insula, the anterior portion appears to be generally more involved during cognitive tasks including language, compared to the other portions (Wager and Barrett 2004; Wager et al. 2004; Zaccarella and Friederici 2015). In addition to the left and right adINS we obtained from Kelly's experiment, we extracted from the HOC atlas two masks covering the left and right FOP, respectively, and another one for the left Fusiform Gyrus. To ensure consistency across the HOC atlas and the Anatomy atlas, we also extracted a mask covering the left BA 44. To note, in any of the SVC analyses, we were not able to find any difference between two-words phrases and two-words lists in the left and right adINS, combined together with the contiguous FOP masks. The same was true for the left and right FOP and for the left Fusiform Gyrus. Crucially, however, we found a significantly active cluster from the comparison between phrases and lists in BA44, which basically reproduced the one we found using the Anatomy Toolbox in the main text (x = –48; y = 17; z = 17; Z = 3.39; cluster = 11 voxels).

References

Andrews, S. (1997). The effect of orthographic similarity on lexical retrieval: Resolving neighborhood conflicts. *Psychon. Bull. Rev.* 4, 439-461.

Coltheart, M., Davelaar, E., Jonasson, J.T., Besner, D. (1977). "Access to internal lexicon", in *Attention and performance IV*,ed., editor. (Hillsdale, New Jersey: Erlbaum), 535-555.

Desikan, R.S., Segonne, F., Fischl, B., Quinn, B.T., Dickerson, B.C., Blacker, D., Buckner, R.L., Dale, A.M., Maguire, R.P., Hyman, B.T., Albert, M.S., Killiany, R.J. (2006). An automated labeling system for subdividing the human cerebral cortex on MRI scans into gyral based regions of interest. *Neuroimage*. 31, 968-980.

Duyck, W., Desmet, T., Verbeke, L.P., Brysbaert, M. (2004). WordGen: a tool for word selection and nonword generation in Dutch, English, German, and French. *Behav. Res. Methods Instrum. Comput*. 36, 488-499.

Holcomb, P.J., Grainger, J., O'Rourke, T. (2002). An electrophysiological study of the effects of orthographic neighborhood size on printed word perception. *J. Cogn. Neurosci.* 14, 938-950.

Kelly, C., Toro, R., Di Martino, A., Cox, C.L., Bellec, P., Castellanos, F.X., Milham, M.P. (2012). A convergent functional architecture of the insula emerges across imaging modalities. *Neuroimage*. 61, 1129-1142.

Wager, T.D., Barrett, L.F. (2004). From affect to control: Functional specialization of the insula in motivation and regulation. *PsychExtra*.

Wager, T.D., Jonides, J., Reading, S. (2004). Neuroimaging studies of shifting attention: a meta-analysis. *Neuroimage*. 22, 1679-1693.

Zaccarella, E., Friederici, A.D. (2015). Reflections of word processing in the insular cortex: a sub-regional parcellation based functional assessment. *Brain Lang.* 142, 1-7.

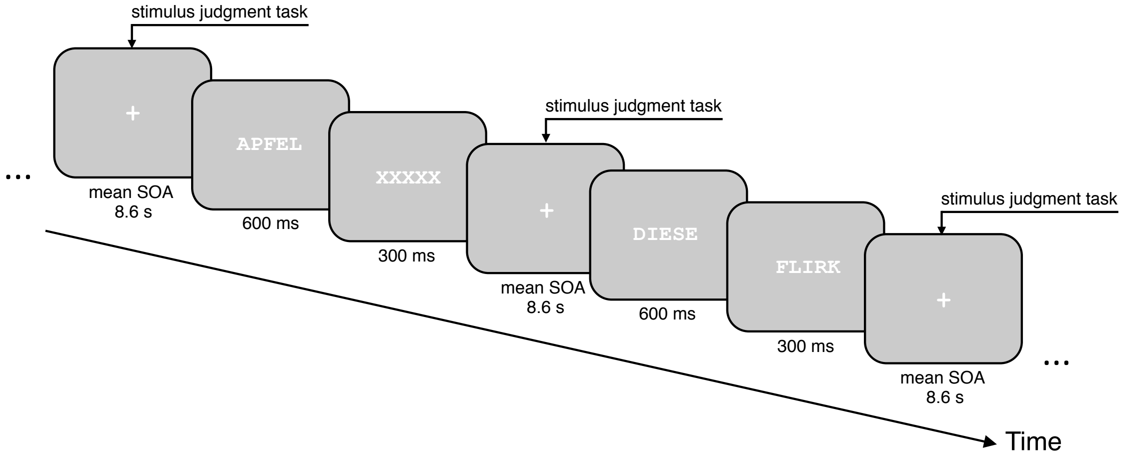

Supplementary Figure 1.
Experimental procedure with timing presentation and task

A single trial consisted of a white fixation cross which remained at the center of the screen until a random jitter of either 0 ms or 1000 ms after volume acquisition started the visual stimulation. Stimulus-onset-asynchrony was 8.6 s on average. All trials had a total duration of 900 ms. Given that our stimulus construction was syllable-constrained, the first bi-syllabic word remained on the screen for 600 ms, while the second monosyllabic word/X string lasted 300 ms. As soon as the fixation cross reappeared, immediately after the second item within the trial had been shown, subjects were requested to indicate via triple-choice button-pressing whether the two words together formed a phrase (e.g. DIESE FLIRK= yes), did not for a phrase, but just a list of two nouns (APFEL, FLIRK= no), or trash trial with X strings (DIESE XXXXX/APFEL XXXXX = trash).

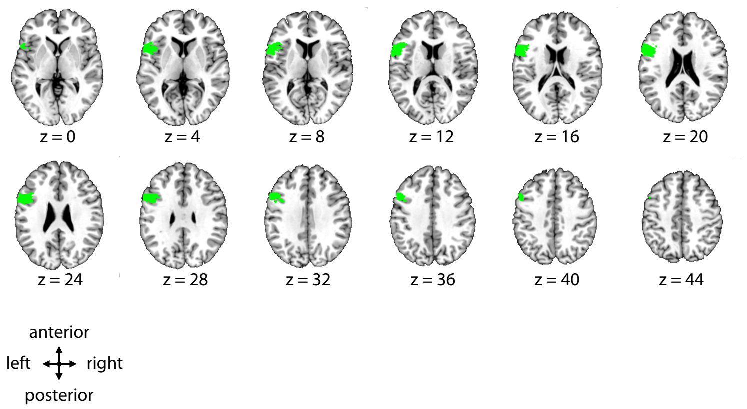

Supplementary Figure 2.
Anatomical region for the Volume-of-Interest Analysis

For the volume-of-interest analysis, BA 44 VOI was defined according to the cytoarchitectonic maps of area 44 of the left hemisphere included in the Anatomy toolbox for SPM.

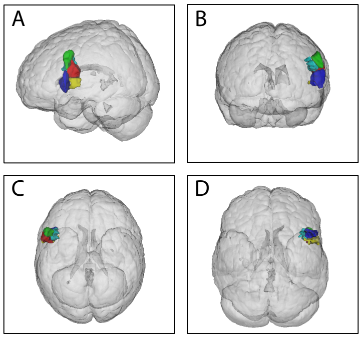

Supplementary Figure 3.
BA44 clusters for the Cluster-of-Interest Analysis

We used the five BA 44 clusters taken from Clos et al. (2013) in the Cluster-of-Interest Analysis, normalized to the MNI space, depicted from four different views (A, sagittal; B, frontal; C, axial; D; ventral view). Cluster 1 (red); Cluster 2 (green); Cluster 3 (blue); Cluster 4 (yellow); and Cluster 5 (cyan). The five clusters are freely available on the Jülich website (http://www.fzjuelich.de/inm/inm1/EN/Forschung/Brain_Network_ Modeling/Brain_Network_Modeling_node.html). Mean signal extraction from the five clusters was done using Marsbar 0.41 for SPM (available at http://marsbar.sourceforge.net).

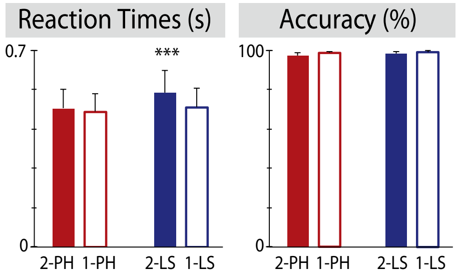

Supplementary Figure 4.
Behavioral results

No significant effect for accuracy was found. A significant effect for structure ($F_{(1,21)} = 25.003$; $p < .0001$) and an interaction between structure and words ($F_{(1,21)} = 5.896$; $p < 0.05$) were found for the reaction time data. A series of paired t-tests revealed that subjects were slower for 2-LS compared to 2-PH ($t = 3.93$; $p < .001$). An almost significant difference for LS: 2>1 was found ($p = .059$), while there was no difference for the contrast PH: 2>1 ($p > .1$).

Anatomical area	MNI-coordinate			K_E	Z_E
	X	Y	Z	(voxels)	
**WORDS (whole-brain)**					
Left FOP/adINS	–33	23	–2	77	6.44
Right FOP/adINS	36	23	–2	38	5.87
Left BA 44/pars opercularis	–48	11	7	67	5.48
additional regions					
Left Fusiform Gyrus	–48	–55	–20	14	5.1
**PHRASE vs. LIST (volume-of-interest, BA 44)**					
**DIESE FLIRK > APFEL FLIRK**					
Left BA 44/pars opercularis	–48	17	16	12	3.65
**APFEL FLIRK > DIESE FLIRK**					
ns.	-	-	-	-	-

Supplementary Table 1.
Functional results

Whole-brain analysis and the small-volume-correction (SVC) analysis in Brodmann Area (BA) 44. Voxel dimension is 3 mm^3. All values are FWE-corrected at $p<0.05$.

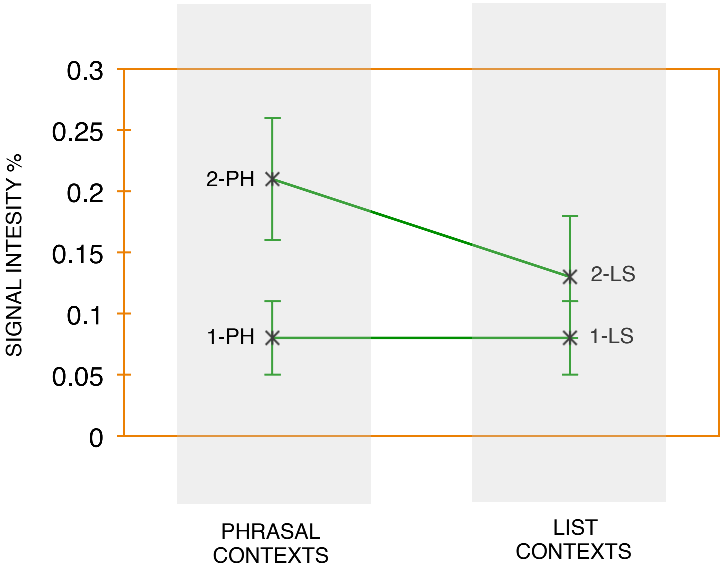

Supplementary Figure 5.
Signal intensity difference with interaction effect in BA44

Prior direct comparison between phrase and list at two-words level, we first run an ANOVA with factors WORDS and STRUCTURE within BA44 to gain information about a possible interaction within the region. We detected within the region the 3D voxel showing the highest peak by downloading the unthresholded WORDS x STRUCTURE activation map we obtained from the SPM group-averaged output (x = –51; y = 20; z = 13). From this 3D coordinates we then extracted signal intensity for all four conditions to verify whether an interaction between WORDS and STRUCTURE would have survived statistical control. To note, we found a significant interaction between the two factors at $p = 0.039$ level ($F_{(1,21)} = 4.83$). Direct comparison between 2-PH and 1-PH was significant at $p < 0.001$ level (t = 3.87), as it was between 2-PH and 2-LS at p = 0.007 level (t = 2.94). Direct comparison between 2-LS and 1-LS was not significant (t = 1.67; p = 0.11). Error bars denote SEM.

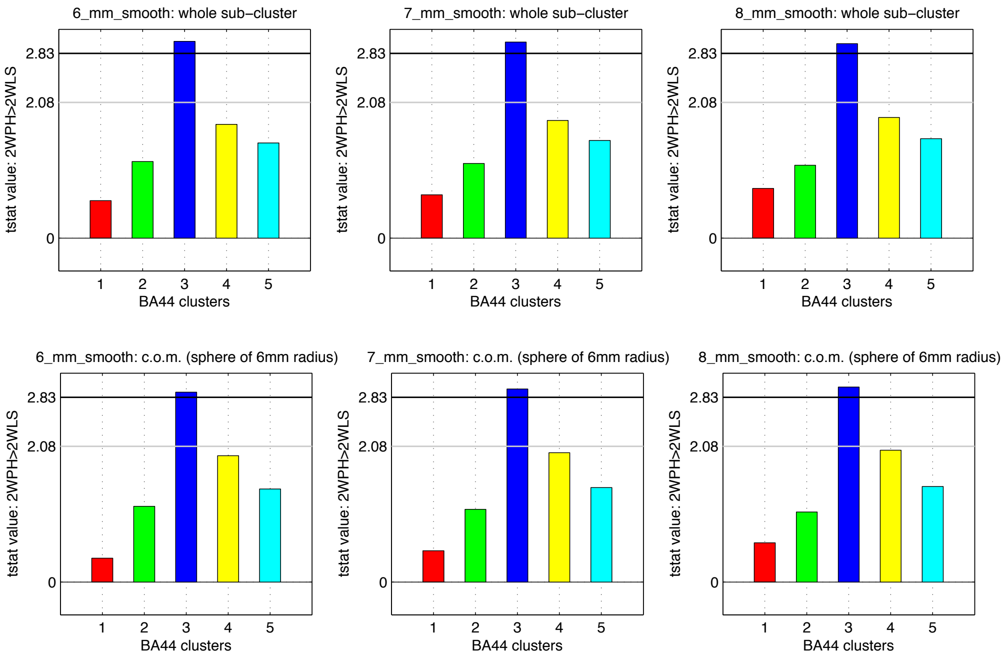

Supplementary Figure 6.
Cluster-of-Interest Analysis with different smoothing kernel and anatomical extensions

To evaluate C3 consistency for merge in BA44, we performed additional signal intensity extraction in the area, for which we varied both smoothing kernel (6mm, left panels; 7mm, middle panels; 8mm, right panels) and anatomical extension of the interested sub-cluster (whole sub-cluster, top panels; center of mass, bottom panels). The unique 3D centers-of-mass for each cluster were obtained using the *c_o_m* function from the Marsbar toolbox, and then transformed into 6mm spheres. A t-test comparison between 2WPHs and 2WLSs for each seed was performed for each BA44 cluster, and the resulting t-stat value reported along the y-axis in each panel. We found a significant difference between 2WPHs and 2WLSs in C3 only, which survived Bonferroni-correction in all of the above cases. To note, the top rightmost panel is a replication of the data reported in Figure 3, included here for completeness. Cluster 1 (red); Cluster 2 (green); Cluster 3 (blue); Cluster 4 (yellow); Cluster 5 (cyan). p = 0.05 (gray line, df = 21); p = 0. 01 (black line; Bonferroni-corrected threshold, df = 21).
